# Gender Differences in the Association between Serum Uric Acid and Prediabetes: A Six-Year Longitudinal Cohort Study

**DOI:** 10.3390/ijerph15071560

**Published:** 2018-07-23

**Authors:** Jia Liu, Zhan Zhao, Yongmin Mu, Xiaoping Zou, Dechun Zou, Jingbo Zhang, Shuo Chen, Lixin Tao, Xiuhua Guo

**Affiliations:** 1 School of Public Health, Capital Medical University, Beijing 100069, China; liujia@ccmu.edu.cn; 2 Beijing Municipal Key Laboratory of Clinical Epidemiology, Capital Medical University, Beijing 100069, China; 3 State Key Lab of Transducer Technology, Institute of Electronics, Chinese Academy of Sciences, Beijing 100049, China; zhaozhan@mail.ie.ac.cn; 4 Institute of Electronics, University of Chinese Academy of Sciences, Beijing 101408, China; 5 Computer Department, Beijing Information Science and Technology University, Beijing 100101, China; yongminmu@163.com (Y.M.); xpzou2014@163.com (X.Z.); 6 Beijing National Laboratory for Molecular Sciences, College of Chemistry and Molecular Engineering, Peking University, Beijing 100871, China; dczou@pku.edu.cn; 7 Department of Information, Beijing Physical Examination Center, Beijing 100077, China; 13910625118@139.com (J.Z.); cs@bjtjzx.com (S.C.)

**Keywords:** serum uric acid, prediabetes, generalized estimating equation, longitudinal cohort study

## Abstract

This study aimed to examine gender differences in the association between serum uric acid (SUA) and the risk of prediabetes in a longitudinal cohort. A total of 8237 participants in the Beijing Health Management Cohort study were recruited and surveyed during 2008–2009, and followed up in 2011–2012 and 2014–2015 surveys. Generalized estimating equation (GEE) models were used to evaluate the association between SUA and prediabetes. Furthermore, subgroup analyses assessed the primary outcome according to status of abdominal obesity, age and status of hypertension. During six years of follow-up, we identified 1083 prediabetes events. The GEE analyses confirmed and clarified the association between SUA and prediabetes (RR = 1.362; 95% CI = 1.095–1.696; *p* = 0.006) after adjusting for other potential confounders, especially in females (RR = 2.109; 95% CI = 1.329–3.347; *p* = 0.002). In addition, this association was stronger in the subgroup of females aged ≥48 years old (RR = 2.384; 95% CI = 1.417–4.010; *p* = 0.001). The risk for prediabetes increased significantly with increasing SUA for females in the Chinese population. This association was strongly confirmed in older females aged ≥48 years old rather than in younger females, which may provide clues for pathogenic mechanisms of gender differences in the association between SUA and prediabetes.

## 1. Introduction

Diabetes is one of the most common metabolic disorders in the world and the prevalence of diabetes and prediabetes in adults has been increasing in recent decades [1]. According to a nationally representative survey in China, more than 10.9% of adults are estimated to have diabetes and more than 35.7% to have prediabetes [2]. Increasing prevalence of diabetes and prediabetes in China comprises a major cause of disease burden.

Prediabetes is a metabolic condition characterized by a higher risk for developing diabetes and its associated complications. About 5–10% of individuals with prediabetes will progress to diabetes every year [3]. According to the China Da Qing Diabetes Prevention Study, 93% of the participants with an impaired glucose tolerance in the absence of intervention eventually developed diabetes after 20-year follow-up [4]. However, studies have observed decreasing risk in transition from prediabetes to diabetes after lifestyle or pharmacological interventions [4,5,6]. Unfortunately, current preventive approaches to prediabetes are not entirely effective in controlling this condition [7]. Early identification of prediabetes might contribute to the prevention of diabetes and therefore further studies are needed to identify modifiable early predictor of prediabetes.

Previous evidence showed that increased levels of serum uric acid (SUA) are associated with the higher risk of diabetes [8]. The relationship between SUA and impaired fasting glucose was also investigated in several prospective studies [9,10,11]. Prediabetes is often associated with abdominal obesity, older age and elevated blood pressure [12,13] and it is unclear whether these related risk factors is important in the relationship between SUA and prediabetes. Both with respect to identifying individuals at high risk accurately and providing clues for exploring the pathogenesis of prediabetes, subgroup analyses of these risk factors could improve stratification of disease risk in populations.

On the other hand, as the end-product of purine nucleotide metabolism in humans, plasma uric acid levels are determined by the metabolisms, the urinary excretion rate and dietary intake of endogenous purines, therefore SUA levels are highly variable [14,15]. However, previous studies have often been based on one measurement of SUA at baseline, regardless of high variability of SUA levels in a long-term follow-up. This may cause misjudgment of the relationship between SUA and prediabetes. In contrast, the generalized estimating equation (GEE) model, which uses repeated measurements of the same set of variables during the follow-up, better reflects the actual value of SUA during follow-up [16,17].

Therefore, we hypothesized that repeated measurements of SUA levels are more reasonable than one SUA measurement at baseline when analyzing the relationship between SUA and prediabetes. The longitudinal cohort study was designed to investigate the association between SUA and prediabetes in Chinese population, and the GEE model can adjust for the inherent correlations between the observations to explore the relationship.

## 2. Materials and Methods

### 2.1. Study Population

The Beijing Health Management Cohort (BHMC) study is a large prospective dynamic cohort study investigating the progression from health to metabolic disorders in individuals from urban areas of northeast China. The participants are either current or retired employees in fixed work environments in Beijing, China. For the purposes of this analysis, the 2008–2009 survey of the BHMC study was used as the starting point, the 2011–2012 survey and the 2014–2015 survey were used as the follow-up visits. Health questionnaire interview, physical examination and fasting laboratory measurements were performed at baseline and every follow-up had the consents of all participants. The study was approved by the Ethics Committee of Capital Medical University (NO: 2013SY26).

Among the 11,245 participants, we excluded those with a previous diagnosis of cardiovascular disease (CVD), cerebrovascular disease or cancer (*n* = 326), with diabetes or prediabetes at baseline (*n* = 1146), without follow-up data (*n* = 1278), if they were taking anti-diabetic medication or with a diagnosis of diabetes at follow-up (*n* = 258). This left us with data for 5471 men and 2766 women. All the participants were free of diabetes, prediabetes, CVD and cancer at the time of enrollment.

### 2.2. Definition of Prediabetes

Prediabetes was defined by the WHO guideline as a fasting plasma glucose level 6.0–6.9 mmol/L or a non-fasting plasma glucose level 7.7–11.1 mmol/L, without the use of anti-diabetic medication. Fasting blood samples were measured using the glucose hexokinase method [18].

### 2.3. Anthropology Measurements and Laboratory Measurements

A health questionnaire interview, physical examination and fasting laboratory measurements were performed at baseline and during follow-up, with the written informed consent of the participants. The general health questionnaire covered educational level, physical activity, smoking status, and alcohol intake status. The physical examination included the measurement of height, weight, waist and blood pressure. The body mass index (BMI) was calculated as weight (kg) divided by height (m) squared. Blood pressure was measured in the right arm of the seated participants using an electronic sphygmomanometer. Three readings each of systolic and diastolic blood pressure were recorded at 1–3 min intervals, and the average of the last two measurements was used for further statistical analysis.

Blood samples were collected from participants after an overnight fast of at least 12 h. Fasting laboratory measurements included fasting plasma glucose (FPG), serum uric acid (SUA), total cholesterol (TC), triglycerides (TG), low-density lipoprotein (LDL), high-density lipoprotein (HDL), white blood cell (WBC), red blood cell (RBC), erythrocyte mean corpuscular volume (MCV), red blood cell distribution width (RDW), platelet count (PLT), mean platelet volume (MPV), platelet distribution width (PDW), gamma-glutamyl transferase (GGT), total bilirubin (TBil), serum total protein (STP), blood urea nitrogen (BUN) and serum creatinine (CREA). Blood samples were measured by enzymatic method using a chemistry analyzer (Beckman LX 20, America) at the central laboratory of the hospital.

### 2.4. Statistical Analysis

To illustrate the distribution characteristics, data were presented as median (25, 75th percentile) for continuous variables or number (%) for categorical variables of interest. The characteristics of the participants were compared among groups. The SUA was categorized into 4 groups (Quartile 1–Quartile 4): ≤P25, >P25 and ≤P50, >P50 and ≤P75, and >P75. The comparisons of characteristics were performed using Kruskal–Wallis tests or Chi-square tests among four groups.

GEE model was reported to fit for the repeat measurement data under the framework of logistic regression model [16,17,19]. It was performed to estimate the relative risks (RRs) and 95% confidence intervals (CIs) of SUA for risk of prediabetes. Simple GEE model was firstly used to select variables associated with prediabetes, then those significant variables were adjusted as the potential confounding factors in the multiple GEE model. SAS software package (Version 9.2; SAS Institute, Chicago, IL, USA) was used for statistical analyses, and *p* < 0.05 was considered statistically significant.

## 3. Results

The characteristics of potential confounding factors grouped by SUA quartiles status at baseline are shown in Table 1. At baseline, the statistically significant differences between SUA quartiles status were observed in all listed variables except for PDW, education level, physical activity and smoking status. Table 2 show the incidence rate of prediabetes events among subjects at four levels (Q1–Q4) separately. Of the 1823 individuals with the highest quartile SUA level, 332 (18.21%) developed prediabetes during the six-year follow-up, while for the individuals with the lowest quartile SUA level, 183 out of 2427 (7.54%) developed to prediabetes.

The adjusted RRs and 95% CIs of SUA for the risk of prediabetes are also shown in Table 2. Compared with the lowest quartile of SUA, the risk of prediabetes became higher in simple GEE model (RR = 2.903; 95% CI = 2.391–3.526; *p* < 0.001) after adjusting for age (Table S1). In multiple GEE model, the association between SUA and the risk of prediabetes remained significant (RR = 1.362; 95% CI = 1.095–1.696; *p* = 0.006) after adjusting for other potential confounding factors. Females in the top quartile (Q4) of SUA had a 2.1-fold greater risk for developing prediabetes (RR = 2.109; 95% CI = 1.329–3.347; *p* = 0.002) using Q1 as reference level after adjusting for other potential confounding factors, while no association was detected in males.

**Figure 1:**
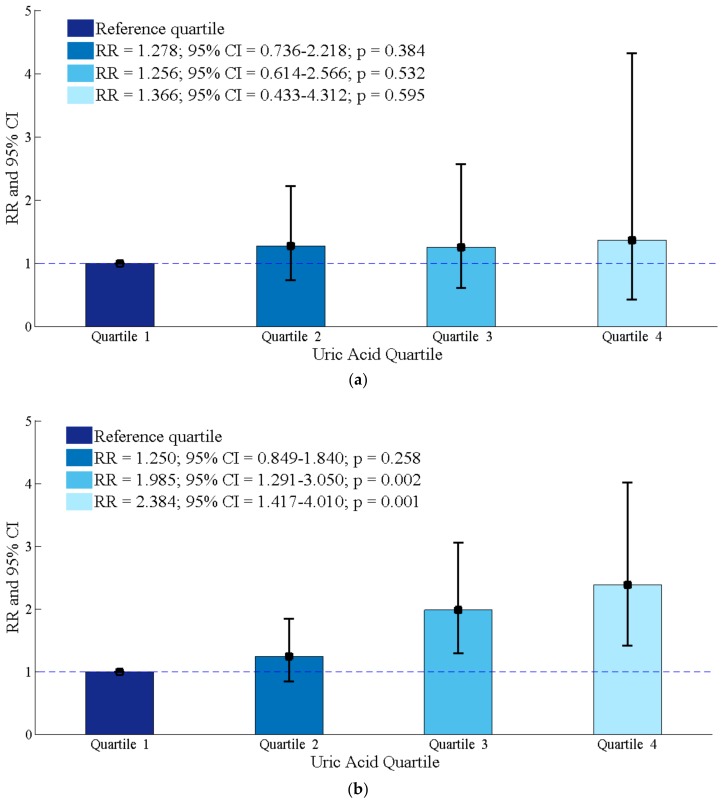
Results of the association between serum uric acid and incident prediabetes in older (age ≥ 48 years old) and younger (age < 48 years old) females. (**a**) Females aged < 48 years old; (**b**) Females aged ≥ 48 years old.

The exclusion of participants with hypertension and participants with abnormal obesity did not change our findings in females (Table S2). There was heterogeneity of the association between SUA and prediabetes in females according to different age group (Figure 1). In older females aged ≥48 years old, the development of prediabetes still increased in relation to the highest quartile of SUA (RR = 2.384; 95% CI = 1.417–4.010; *p* = 0.001), compared with the reference group after adjustments for other potential confounders, but the similar trend was not observed in younger females aged <48 years old.

A comparison of the baseline characteristics between participants and nonparticipants is shown in Table S3. In general, we found that nonparticipants were younger, were more likely to drink alcohol, had slightly higher HDL, WBC, RBC, SBP, DBP, and FPG, had higher PLT and PDW, and had slightly lower TC, TG, LDL, MCV, RDW, GGT, BUN, waist, SBP and SUA.

## 4. Discussion

In this prospective cohort study, we found a significant association between increasing SUA levels and a higher risk of prediabetes, especially in older females aged ≥48 years old. About 20 percent of females with the top quartile (Q4) SUA level developed into prediabetes during the six-year follow-up in our study, making this a high-risk group for prediabetes.

Conflicting results regarding gender differences in the association between SUA and incident prediabetes have been reported [9,10,20]. The Rotterdam Study and a similar study in Japanese demonstrated that elevated SUA predicted prediabetes only in females [9,20]. While the Kailuan study reported that low or high SUA concentrations were both associated with a higher risk of developing impaired fasting glucose only in males. There is relevant evidence reporting a greater increase in glucose-related risk with the higher level of SUA in females. Not only does SUA associate with glucose-related endpoint especially in females, but also hyperglycemia was an independent predictor of metabolic abnormalities and cardiovascular events exclusively in females [21,22,23]. In our study, we found a strong positive association between SUA and incident prediabetes in females rather than in males using the multiple GEE model. Furthermore, exclusion of participants with hypertension or participants with abnormal obesity did not change this association in females. Combining the results from this study and above, it seems reasonable to suppose that the association between SUA and incident prediabetes is more prominent in females than that in males. Therefore, more attention should be paid to the female population with higher level of SUA in the clinical guideline.

Although the link between SUA and prediabetes is more consistent in females, the evidence supporting this association is still unclear. One possible explanation lies in the estrogen and its clinical impact on females. We hypothesize that sex hormones may play a role in the relationship between SUA and prediabetes. According to a study in Chinese city women, the average menopausal age was 48 years old [24]. We therefore selected 48 as the cut-off point for subgroup analysis stratified by age in females. In our study, older females aged ≥48 years old with the top quartiles (Q4) SUA level were two times more likely to have prediabetes, compared with the reference group after adjustments for potential confounding factors (Figure 1), but the similar trend was not observed in females aged <48 years old.

Estrogen is well known to be cardioprotective and antihyperlipidemic [25]. A finding about transsexual persons reported that the male-to-female transsexuals presented a reduction in levels of SUA and an increase in fractional excretion of SUA after one year of treatment with hormone therapy [26]. Several investigators have also demonstrated that SUA levels increase in postmenopausal females, but decrease when hormone therapy is administered [27,28]. These data support that estrogen promotes excretion of SUA and the increased SUA in older females may be explained by the deficiency of estrogens.

Uric acid can promote oxidative damage through decreasing nitric oxide degeneration, followed by inflammation and endothelial dysfunction, which eventually results in insulin resistance [29]. Additionally, vascular endothelial function has been reported to decline with the estrogen deficiency [30]. Furthermore, uric acid can also cause function inhibition of pancreatic beta cells [31], and beta cell function has been reported to decline at the low point of fasting glucose within the normoglycemic range [32]. These findings may support the postulate that decreased estrogen levels may lead to increased uric acid levels, which in turn may cause endothelial dysfunction, predisposing older females to development of insulin resistance. A study reported that the development of diabetes increased with the higher levels of SUA in postmenopausal females, but not in premenopausal females [33]. In our study, the development of prediabetes increased with the higher quartiles of SUA only in older females aged ≥ 48 years old. Our findings suggest that higher SUA levels may associate with the early onset of diabetes.

Our study has several strengths. GEE model is firstly used to evaluate the relationship between the SUA and prediabetes. The association between increasing SUA levels and the risk for prediabetes is adjusting for a relatively wide range of biological covariates. Finally, our study provides evidence for the association between increasing SUA levels and the risk for prediabetes in females, especially in older females aged ≥48 years old. However, several limitations of the current study need to be addressed. Firstly, nutritional data was not collected in our study; this might have an effect on the association between SUA and prediabetes. Secondly, this study has been conducted on a sample of Beijing population and our findings may not be applicable to other populations. Last but not least, because information about behaviors were self-reported, measurement errors are inevitable.

## 5. Conclusions

In summary, our findings showed that SUA was associated with the risk of incident prediabetes in females. However, this association was strongly confirmed in older females aged ≥48 years old rather than in younger females. Further studies need to be conducted to explore the pathogenic mechanisms of this association. This study suggests that attention should be paid to females with higher SUA levels to prevent prediabetes.

## Figures and Tables

**Table 1:** Distribution of potential confounding factors of participants grouped by serum uric acid (SUA) quartiles status at baseline.

Variables	Uric Acid Quartile				*p* Value
	Quartile 1 (*n* = 2427)	Quartile 2 (*n* = 2028)	Quartile 3 (*n* = 1959)	Quartile 4 (*n* = 1823)	
Age (years)	44 (36–53)	47 (38–58)	48 (39–61)	49 (39–61)	<0.0001
TC (mmol/L)	4.55 (4.00–5.13)	4.69 (4.13–5.30)	4.82 (4.24–5.42)	4.91 (4.33–5.56)	<0.0001
TG (mmol/L)	0.95 (0.70–1.31)	1.23 (0.88–1.78)	1.50 (1.06–2.13)	1.75 (1.27–2.57)	<0.0001
LDL (mmol/L)	2.78 (2.31–3.31)	2.98 (2.47–3.53)	3.12 (2.60–3.66)	3.18 (2.68–3.74)	<0.0001
HDL (mmol/L)	1.42 (1.24–1.66)	1.29 (1.11–1.48)	1.21 (1.07–1.39)	1.17 (1.03–1.36)	<0.0001
WBC (10^9^/L)	5.30 (4.50–6.16)	5.60 (4.80–6.69)	5.70 (4.80–6.80)	5.90 (5.08–6.94)	<0.0001
RBC (10^12^/L)	4.32 (4.07–4.62)	4.58 (4.27–4.92)	4.70 (4.39–5.03)	4.72 (4.42–5.02)	<0.0001
MCV (fL)	90.50 (87.30–93.70)	91.60 (88.20–95.1)	91.60 (88.09–95.10)	92.00 (88.80–95.33)	<0.0001
RDW (%)	11.90 (11.20–12.70)	12.10 (11.11–14.90)	12.12 (11.02–15.90)	12.30 (11.30–16.60)	<0.0001
PLT (10^9^/L)	201 (172–236)	194 (165–226)	192 (163–226)	189 (159–221)	<0.0001
MPV (fL)	8.20 (7.60–8.90)	8.20 (7.66–8.90)	8.20 (7.60–8.90)	8.33 (7.70–8.90)	0.0165
PDW (%)	8.30 (7.70–10.30)	8.30 (7.60–10.25)	8.40 (7.60–10.50)	8.40 (7.70–10.25)	0.8953
GGT (U/L)	14.10 (10.70–22.00)	20.60 (14.10–33.20)	24.90 (17.30–41.60)	31.30 (21.00–52.90)	<0.0001
TBIL (μmol/L)	12.90 (10.10–16.50)	14.60 (11.40–18.00)	15.20 (12.09–18.50)	15.50 (12.30–18.80)	<0.0001
STP (g/L)	72.20 (69.40–75.13)	72.40 (69.60–75.17)	72.40 (69.80–75.40)	73.02 (70.30–75.86)	<0.0001
BUN (mmol/L)	4.71 (3.90–5.63)	5.12 (4.36–5.98)	5.36 (4.61–6.17)	5.44 (4.66–6.33)	<0.0001
CREA (umol/L)	74.40 (68.50–82.70)	85.85 (78.15–93.40)	90.60 (84.00–97.30)	94.80 (88.10–102.40)	<0.0001
BMI	23.33 (21.19–25.59)	24.84 (22.81–26.89)	25.95 (23.89–28.02)	26.62 (24.66–28.70)	<0.0001
Waist (cm)	79 (73–85)	86 (80–93)	90 (84–96)	93 (88–98)	<0.0001
SBP (mm Hg)	110 (100–120)	118 (110–130)	120 (110–130)	120 (110–130)	<0.0001
High school or higher education (%)	1894 (94.75)	1608 (94.64)	1577 (93.7)	1449 (94.46)	0.5298
Regular physical activity (%)	701 (35.07)	609 (35.84)	596 (35.41)	567 (36.96)	0.6879
Smoking (%)	201 (10.06)	195 (11.48)	199 (11.82)	175 (11.41)	0.3233
Alcohol drinking (%)	215 (10.76)	255 (15.01)	260 (15.45)	255 (16.62)	<0.0001

Abbreviations: TC = total cholesterol; TG = triglycerides; LDL = low-density lipoprotein; HDL = high-density lipoprotein; WBC = white blood cell; RBC = red blood cell; MCV = erythrocyte mean corpuscular volume; RDW = Red blood cell distribution width; PLT = platelet count; MPV = mean platelet volume; PDW = platelet distribution width; GGT = gamma-glutamyl transferase, TBIL = total bilirubin; STP = serum total protein; BUN = blood urea nitrogen; CREA = serum creatinine; BMI = body mass index; SBP = systolic blood pressure.

**Table 2:** Results of generalized estimating equation (GEE) analysis for serum uric acid (SUA) and prediabetes with their relative risks (RRs) and 95% confidence intervals (CIs).

Uric Acid Quartile	*n*	Prediabetes Events	Incidence Rate	RR (95%CI)	Multivariate-Adjusted		
					RR	95% CI	*p* Value
all ^1^				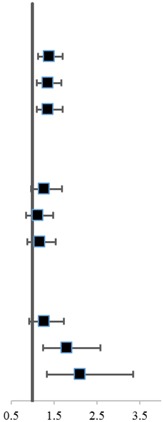
Q1	2427	183	7.54%	
Q2	2028	248	12.23%		1.388	1.135–1.698	0.001
Q3	1959	320	16.33%		1.357	1.097–1.678	0.005
Q4	1823	332	18.21%		1.362	1.095–1.696	0.006
male ^2^							
Q1	632	68	10.76%				
Q2	1398	190	13.59%		1.272	0.963–1.681	0.091
Q3	1705	279	16.36%		1.118	0.846–1.477	0.434
Q4	1736	311	17.91%		1.161	0.877–1.537	0.297
female ^3^							
Q1	1795	115	6.41%				
Q2	630	58	9.21%		1.266	0.925–1.731	0.140
Q3	254	41	16.14%		1.792	1.241–2.588	0.002
Q4	87	21	24.14%		2.109	1.329–3.347	0.002

^1^ The association between SUA and prediabetes was assessed by multiple GEE analysis with age, TC, TG, HDL, WBC, RBC, MCV, RDW, PLT, MPV, PDW, GGT, STP, SBP and waist adjustment in all participants. ^2^ The association between SUA and prediabetes was assessed by multiple GEE analysis with age, TC, TG, HDL, WBC, RBC, MCV, RDW, PLT, MPV, PDW, GGT, STP, SBP and waist adjustment in male. ^3^ The association between SUA and prediabetes was assessed by multiple GEE analysis with age, TC, TG, HDL, WBC, RBC, RDW, PLT, MPV, PDW, GGT, STP, SBP, BMI and waist adjustment in female.

## Supplementary Materials

The following are available online at http://www.mdpi.com/1660-4601/15/7/1560/s1, Table S1: Results of age-adjusted generalized estimating equation (GEE) analysis for serum uric acid (SUA) and prediabetes with their risk ratio (RR) and 95% confidence intervals (CI); Table S2: Results of subgroup analyses for the association between serum uric acid (SUA) and incident prediabetes in female; Table S3: Characteristics comparison between participants and nonparticipants.

## Abbreviations

SUA	serum uric acid
GEE	generalized estimating equation
BHMC	Beijing Health Management Cohort
CVD	Cardiovascular disease
BMI	Body mass index
FPG	Fasting plasma glucose
TC	Total cholesterol
TG	Triglycerides
LDL	Low-density lipoprotein
HDL	High-density lipoprotein
WBC	White blood cell
RBC	Red blood cell
MCV	Erythrocyte mean corpuscular volume
RDW	Red blood cell distribution width
PLT	Platelet count
MPV	Mean platelet volume
PDW	Platelet distribution width
GGT	Gamma-glutamyl transferase
TBil	Total bilirubin
STP	Serum total protein
BUN	Blood urea nitrogen
CREA	Serum creatinine
