# Supplementary material for: Gender Differences in the Association between Serum Uric Acid and Prediabetes: A Six-Year Longitudinal Cohort Study
Source: Int J Environ Res Public Health. 2018 Jul 23;15(7):1560. doi: 10.3390/ijerph15071560 (PMC6068609; doi:10.3390/ijerph15071560)
Supplement: Supplementary file 1 [file ijerph-15-01560-s001.zip › supplementary table2.docx]

**Table S2.** Results of subgroup analyses for the association between serum uric acid (SUA) and incident prediabetes in female.

|  | Age-adjusted | | |  | Multivariate-adjusted | | |
| --- | --- | --- | --- | --- | --- | --- | --- |
|  | RR | 95 % CI | *P* value |  | RR | 95 % CI | *P* value |
| Exclusion of participants with hypertension in female |  |  |  |  |  |  |  |
| Q1 | ref | ref | ref |  | ref | ref | ref |
| Q2 | 1.687 | 1.189-2.394 | 0.003 |  | 1.071 | 0.746-1.537 | 0.710 |
| Q3 | 3.421 | 2.292-5.105 | <0.001 |  | 1.759 | 1.158-2.670 | 0.008 |
| Q4 | 4.307 | 2.590-7.163 | <0.001 |  | 2.102 | 1.245-3.548 | 0.005 |
| Exclusion of participants with abnormal obesity in female |  |  |  |  |  |  |  |
| Q1 | ref | ref | ref |  | ref | ref | ref |
| Q2 | 0.853 | 0.497-1.464 | 0.564 |  | 0.548 | 0.319-0.940 | 0.029 |
| Q3 | 2.185 | 1.124-4.248 | 0.021 |  | 1.058 | 0.505-2.217 | 0.881 |
| Q4 | 4.754 | 1.915-11.80 | <0.001 |  | 2.544 | 1.074-6.023 | 0.034 |

^1^ The association between SUA and prediabetes was assessed by multiple GEE analysis with age, TC, TG, LDL, HDL, WBC, RBC, RDW, PLT, MPV, PDW, GGT, STP, SBP, BMI and waist adjustment in female without hypertension.

^2^ The association between SUA and prediabetes was assessed by multiple GEE analysis with age, TG, HDL, WBC, RBC, RDW, PLT, MPV, PDW, SBP, BMI and waist adjustment in female without abnormal obesity.
