# Supplementary material for: Gender Differences in the Association between Serum Uric Acid and Prediabetes: A Six-Year Longitudinal Cohort Study
Source: Int J Environ Res Public Health. 2018 Jul 23;15(7):1560. doi: 10.3390/ijerph15071560 (PMC6068609; doi:10.3390/ijerph15071560)
Supplement: Supplementary file 1 [file ijerph-15-01560-s001.zip › supplementary table3.docx]

**Table S3.** Characteristics comparison between participants and nonparticipants.

| **Variable** | **Participants (8237)** | **Nonparticipants (1278)** |
| --- | --- | --- |
| Age (years) | 47(37-58) | 46(36-54) * |
| TC (mmol/L) | 4.72(4.15-5.35) | 4.62(4.05-5.23) * |
| TG (mmol/L) | 1.29(0.89-1.91) | 1.21(0.83-1.79) * |
| LDL (mmol/L) | 3.01(2.48-3.55) | 2.79(2.26-3.38) * |
| HDL (mmol/L) | 1.28(1.1-1.5) | 1.34(1.14-1.56) * |
| WBC (10^9^/L) | 5.6(4.76-6.6) | 5.69(4.9-6.72) * |
| RBC (10^12^/L) | 4.56(4.25-4.91) | 4.67(4.35-5.01) * |
| MCV (fL) | 91.38(88-94.8) | 89.7(86.94-92.19) * |
| RDW (%) | 12.1(11.2-14.3) | 10.27(9.2-11.6) * |
| PLT (10^9^/L) | 194.94(165-228) | 210.63(178.44-245.02)* |
| MPV (fL) | 8.3(7.66-8.9) | 8.32(7.81-8.8) |
| PDW (%) | 8.3(7.7-10.29) | 12.25(10-14) * |
| GGT (U/L) | 21.6(13.7-36.57) | 19.9(13.6-31.5) * |
| TBIL (μmol/ L ) | 14.5(11.3-18.04) | 14.04(10.9-18) |
| STP (g/L) | 72.5(69.8-75.4) | 72.51(69.87-75.5) |
| BUN (mmol/L) | 5.16(4.34-6.03) | 5.04(4.22-5.98) * |
| CREA (umol/L) | 86.7(76.5-95.1) | 85.9(75.6-95.2) |
| BMI | 25.08(22.86-27.38) | 24.78(22.51-27.5) |
| Waist (cm) | 87(79.6-94) | 84.94(77.75-92.79) * |
| SBP (mm Hg) | 118.27(110-130) | 116(108-125) * |
| High school or higher education (%) | 6528(94.4) | 926(93.44) |
| Regular physical activity (%) | 2473(35.76) | 371(37.44) |
| Smoking (%) | 770(11.14) | 108(10.9) |
| Alcohol drinking (%) | 985(14.24) | 166(16.75) * |

* *P* < 0.05.

Abbreviations: TC = total cholesterol; TG = triglycerides; LDL =low-density lipoprotein; HDL = high-density lipoprotein; WBC = white blood cell; RBC= red blood cell; MCV= erythrocyte mean corpuscular volume; RDW = Red blood cell distribution width; PLT = platelet count; MPV = mean platelet volume; PDW = platelet distribution width; GGT = gamma-glutamyl transferase, TBIL = total bilirubin; STP = serum total protein; BUN = blood urea nitrogen; CREA = serum creatinine; BMI = body mass index; SBP = systolic blood pressure.
