# Supplementary material for: Gender Differences in the Association between Serum Uric Acid and Prediabetes: A Six-Year Longitudinal Cohort Study
Source: Int J Environ Res Public Health. 2018 Jul 23;15(7):1560. doi: 10.3390/ijerph15071560 (PMC6068609; doi:10.3390/ijerph15071560)
Supplement: Supplementary file 1 [file ijerph-15-01560-s001.zip › supplementary table1.docx]

**Table S1.** Results of age-adjusted generalized estimating equation (GEE) analysis for serum uric acid (SUA) and prediabetes with their risk ratio (RR) and 95% confidence intervals (CI).

|  | RR | 95 % CI | *P* value |
| --- | --- | --- | --- |
| All population |  |  |  |
| Q1 | ref | ref | ref |
| Q2 | 1.923 | 1.587-2.332 | <0.001 |
| Q3 | 2.280 | 1.873-2.775 | <0.001 |
| Q4 | 2.903 | 2.391-3.526 | <0.001 |
| Male population |  |  |  |
| Q1 | ref | ref | ref |
| Q2 | 1.353 | 1.034-1.771 | 0.027 |
| Q3 | 1.355 | 1.036-1.771 | 0.026 |
| Q4 | 1.752 | 1.345-2.282 | <0.001 |
| Female population |  |  |  |
| Q1 | ref | ref | ref |
| Q2 | 1.936 | 1.433-2.615 | <0.001 |
| Q3 | 3.409 | 2.407-4.826 | <0.001 |
| Q4 | 4.177 | 2.688-6.490 | <0.001 |
